# Predicting the potential distribution and forest impact of the invasive species *Cydalima perspectalis* in Europe

**DOI:** 10.1002/ece3.7476

**Published:** 2021-03-26

**Authors:** Quim Canelles, Emili Bassols, Jordi Vayreda, Lluís Brotons

**Affiliations:** ^1^ InForestJru (CREAF‐CTFC) Solsona Spain; ^2^ Parc Natural de la Zona Volcànica de la Garrotxa Olot Spain; ^3^ CREAF Cerdanyola del Vallès Spain; ^4^ CSIC Cerdanyola del Vallès Spain

**Keywords:** Biological invasion, box tree moth, ecological niche model, forest disturbance, habitat suitability

## Abstract

Invasive species have considerably increased in recent decades due to direct and indirect effects of ever‐increasing international trade rates and new climate conditions derived from global change. We need to better understand how the dynamics of early species invasions develop and how these result in impacts on the invaded ecosystems. Here we studied the distribution and severe defoliation processes of the box tree moth (*Cydalima* *perspectalis* W.), a tree defoliator insect native to Asia and invasive in Europe since 2007, through the combination of species distribution models based on climate and landscape composition information. The results showed that the combination of data from the native and the invaded areas was the most effective methodology for the appropriate invasive species modeling. The species was not influenced by overall landscape factors, but only by the presence of its host plant, dispersal capacity, and climate suitability. Such climate suitability was described by low precipitation seasonality and minimum annual temperatures around 0°C, defining a continentality effect throughout the territory. We emphasize the need of studying distribution and severe defoliation processes separately because we identified that climate suitability was slightly involved in limiting species spread processes but strongly constrained ecosystem impact in terms of defoliation before the species reaches equilibrium with the new environment. New studies on habitat recovery after disturbance, ecological consequences of such impact, and community dynamics in a context of climate change are required for a better understanding of this invasive species.

## Graphical abstract

Distribution and severe defoliation processes of the box tree moth (*Cydalima perspectalis* W.), a tree defoliator insect native to Asia and invasive in Europe, were studied. We identified the main determinants that influence the species climatic and ecological niche, as well as we projected the distribution and impact to the invaded areas.

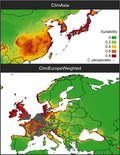

## 1 INTRODUCTION

The incidence of alien species to invaded host ecosystems has increased in recent years due to climate change and the growth in international trade (Hulme, 2003). The movement of alien species (not native to a specific location, also referred to as introduced or non‐native species) has been linked to human activity for millennia due to international trade that favors the accidental introduction of species into new ecosystems (Bradshaw et al., 2016; Hulme, 2009). The association is so strong that key moments in history involving international commerce match alien species redistribution peaks, highlighting the end of the Middle Ages, the industrial revolution, and, most remarkably, the recent era of market globalization (Hulme, 2009). The last 50 years have been characterized by an increase in transport networks and demand for commodities, allowing displacement of species into new ecosystems. This trend is more notable in countries with high economic activity, large transport infrastructure, and facilitators of international commerce (such as dismantling of customs checkpoints in the case of European Union countries; Hulme, 2009; Roques et al., 2016).

Global warming is known to push changes in species distributional areas and may increase the probability that alien species become established and markedly impact new locations, becoming invasive species (alien species with an ecological or healthy impact). Many invasive species share traits such as short generation times, rapid dispersal, or environmental plasticity that could be advantageous in a transitioning climate (Dukes & Mooney, 1999). Moreover, climate change may intensify the impact severity and likelihood of pest outbreaks, both in native and alien species (Bebber et al., 2013; FAO, 2008). However, some scientists question the relevance of climate change on alien species performance, because its importance is equivalent to other factors such as land cover or geography of each site (Pyšek et al., 2010). They also argue that new climate conditions do not always lead to increasing the climate niche of alien species, because each species could perform differently in distinct areas (Bebber et al., 2013; Peterson et al., 2008).

Biological invasions progress through the following consecutive phases: (a) arrival, (b) establishment, (c) dispersion, and (d) impact (Vermeij, 1996). Only a small percentage of alien species eventually progress to the last stage when the species is considered invasive and causes damage to the environment, human economy, or human health (Roques et al., 2016). However, their effects on the economy, society, and ecology may be massive. Invasive insects (the second largest invasive group behind vascular plants; Seebens et al., 2017) are mainly studied because they cause economic and social damage in agriculture. The damage caused by invasive insect species in the forest industry is also significant, reaching $70 billion annually for the world (Bradshaw et al., 2016).

The ecological effects of invasive insect species occur at different levels of biological organization, from genetics (genetic hybridization with native species) to populations (new prey–predator relations) to ecosystems (phylogenic and taxonomic diversity, trophic networks, ecosystem productivity, etc.; Blackburn et al., 2014). Landscape may be directly affected by massive cases of herbivory leading plant species or entire ecosystems to high risk (e.g., the case of *Elatobium* *abietinum* W., which is invasive in North America and threatening to *Picea* *engelmannii*; Lynch, 2004). However, invasive insect species can also have indirect effects on communities, either by frustrating plant reproduction mechanisms (the case of *Lymantria* *dispar* L. causing oak seedling mortality both in native and invaded area; Gottschalk, 1989) or by triggering a cascade effect leading to the facilitation of other disturbances such as fire, arrival of other diseases, or sensitivity to drought (Canelles et al., 2021).

Management actions and policies are required to deal with invasive species impacts on ecosystem functioning and associated goods and services. Identifying the potential distribution and the stage of invasion of the studied species is critical for an adequate management strategy, and quick development of action plans is required because eradication after the species establishment stage represents colossal costs and is often unsuccessful (Perrings et al., 2005; Pimentel et al., 2005). Species distribution models (SDMs) are commonly used to predict the potential distribution range of invasive species (Uden et al., 2015). SDMs are built on the general concept of a fundamental niche and predicted species distributions that depend on the modeling algorithm that is applied. Such algorithms are trained with a given species occurrence and associated environmental data and then projected onto different areas to identify regions with environmental suitability for such species (Elith & Leathwick, 2009; Guisan et al., 2017). However, the application of species distribution models to invasive species (SDMi) is under discussion because it may contradict two SDM assumptions: (a) ecological niches are stable in space and time, and (b) the studied species is in quasi‐equilibrium with the environment (Barbet‐Massin et al., 2018; Elith & Graham, 2009; Gallien et al., 2010, 2012). Thus, for an adequate understanding of the species, it is necessary to combine information from the original area and the invaded area as well as develop a synoptic view of the mechanisms involved in the invasion process, identify source populations, clarify invasive pathways, and describe the main ecological factors and processes that favor or restrict invasive species performance (Bras et al., 2019; Kriticos et al., 2003; Pyšek et al., 2010). Moreover, differential description of species distribution (where the species is present) and severe damage dynamics (where the impact is high) may help accurate management strategies (Roques et al., 2016). To perform proper management, approaches must combine global and regional information of the invasive species as well as incorporate its processes dynamically (Hulme, 2003). Thus, cooperation between scientific researchers and managers is mandatory.

**FIGURE 1:**
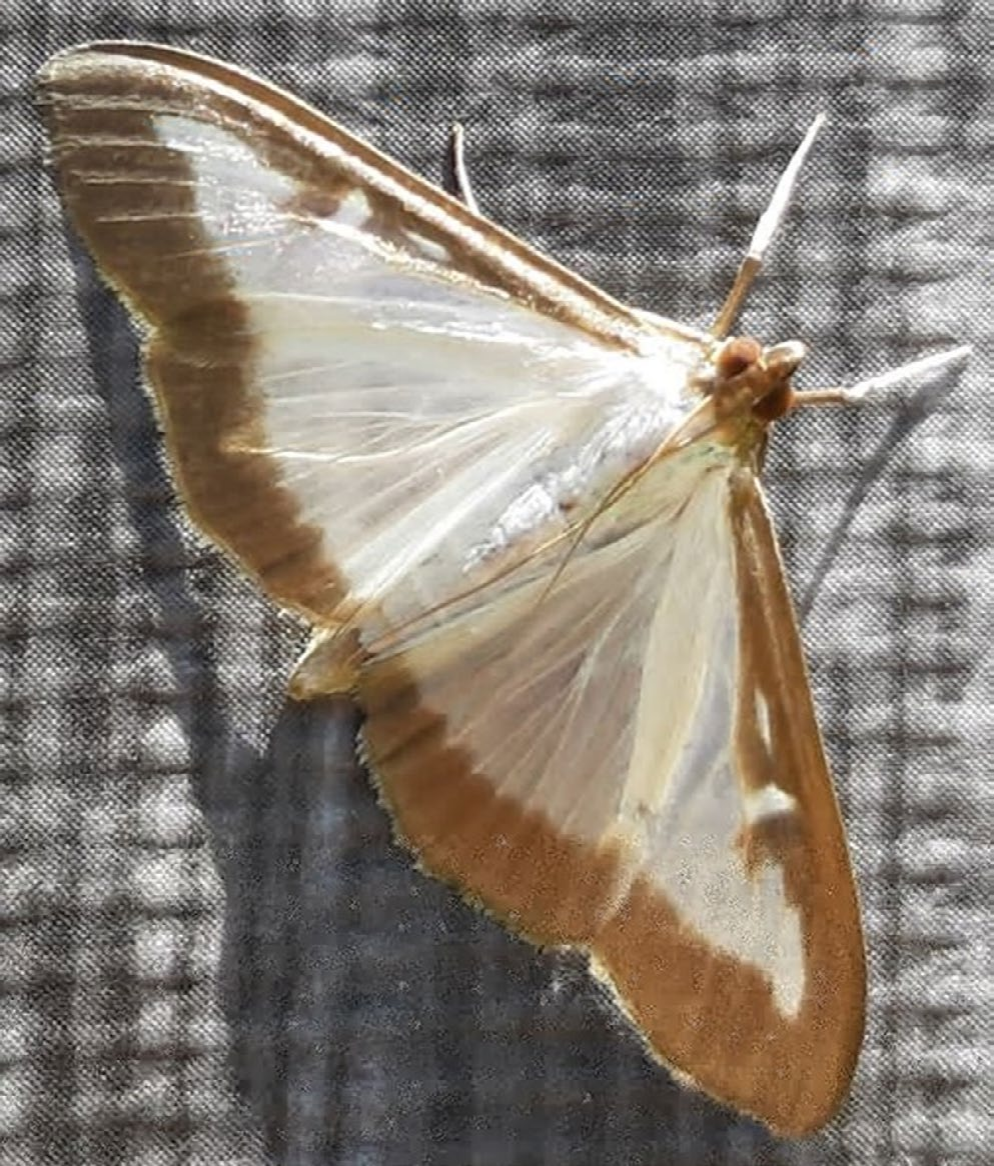
Adult individual of *Cydalima* *perspectalis* W. Source: Jenny Llopis

The box tree moth, *Cydalima* *perspectalis* W. (Figure 1), native to Asia, is a prime example of an invasive alien species in Europe with a potential for high ecosystem impact. This species arrived in central Europe in 2007 and has spread throughout the continent (Kim & Park, 2013; Krüger, 2008; Maruyama & Shinkaji, 1987). *Cydalima* *perspectalis* causes economic and huge ecological effects because during its caterpillar phase it feeds on species of the genus *Buxus*, often leading to complete defoliation and producing significant damage to natural habitats as well as parks and gardens with ornamental boxwood fences. The persistence of consecutive defoliation episodes and the habit of caterpillars to eat the bark when there are no leaves may lead to the death of the plant (Artola, 2019; Mitchell et al., 2018; Straten & Muus, 2010). The ecological effects of *C*. *perspectalis* and consequent boxwood decay include the risk of species hosted in box trees (Mitchell et al., 2018) and the endangerment of some Mediterranean mountain ecosystems where the box tree is the main undercover species (Di Domenico et al., 2012).

In this study, we aimed to identify the main determinants of *C*. *perspectalis* distribution and how these factors constrain distribution dynamics and ecosystem impact after the species establishment in a new environment. More specifically, we addressed the following questions: (a) Does the climate niche in the native area define the expansion process of the species in recently invaded areas? (b) Is the species distribution influenced by habitat composition (e.g., forest cover, habitat fragmentation) interacting with the climate niche? (c) Are the processes of spread and impact different in terms of the underlying factors that determine these dynamics?

## 2 MATERIALS AND METHODS

### 2.1 Study species

The tree box moth *C*. *perspectalis* W (Lepidoptera: Crambidae), formerly placed in the genera *Glyphodes*, *Diaphania*, and *Neoglyphodes* (Mally & Nuss, 2010), is a native insect from Asia, mainly China, Japan, Korea, and India (Kim & Park, 2013; Maruyama & Shinkaji, 1987; Park, 2008). This species was first introduced in Europe in 2007, entering in Germany via the trade of ornamental box trees between China and Europe (Casteels et al., 2011; Leuthardt, 2013; Nacambo et al., 2014). In recent years, new observations have been recorded in Switzerland and the Netherlands (Leuthardt, 2013; Straten & Muus, 2010), and in a period of 10 years, the insect has spread all across Europe and into Asia Minor (Bras et al., 2019). Such spread is thought to be due to the flight capability of the species but mainly due to the accidental transport of individuals to different localities in Europe (Kenis et al., 2009; Matošević, 2013; Roques et al., 2016). According to its biological development, *C*. *perspectalis* has a classical egg–caterpillar–pupa–adult cycle. Adult mating phases start in the spring and end in the late summer with a cycle of 2–3 generations per year (Bakay & Kollár, 2018; Leuthardt, 2013; Nacambo et al., 2014; Nagy et al., 2017; Santi et al., 2015), but up to 5 generations per year have been reported in China (Chen et al., 2005). Temperature (minimum temperature and degree days) and relative humidity have usually been considered critical for species development (Suppo et al., 2020). Egg hatching success has been reported successful between 15 and 30ºC in Asia (Maruyama & Shinkaji, 1987), while development threshold temperatures and degree days (dd) were 10.9°C and 45 dd for the egg stage; 8.4°C and 322 dd for the larval stage; and 11.5°C and 133 dd for the pupal stage (Nacambo et al., 2014). In its native range, up to 10 different *Buxus* species have been documented as host plants, with a preference for *Buxus* *microphylla* Siebold & Zucc. (Maruyama & Shinkaji, 1991). In Europe, *C*. *perspectalis* is hosted on *Buxus* *sempervirens* L., a common species in southern and Western European forests as well as a decorative plant in gardens throughout the continent (Artola, 2019; Bras et al., 2019; Wan et al., 2014).

### 2.2 Study area

The area of study was Catalonia, a 32,114 km^2^ region located in northeastern Spain with a predominantly Mediterranean climate. Its complex orography, with an altitude range from 0 to more than 3,000 m above sea level (a.s.l.) and a coast extending over 750 km, has resulted in a highly diverse climate. The region is extensively covered by forest (40%) and scrublands (16%), while agricultural lands (29%) and urban areas (6%) contribute to the fragmentation of natural areas (Ibàñez et al., 2002). Forests are dominated by Aleppo pine (*Pinus* *halepensis* M.), Holm oak (*Quercus* *ilex* L.), Scots pine (*Pinus* *sylvestris* L.), and European black pine (*Pinus* *nigra* subsp. *salzmannii* A.), which together represent two‐thirds of the total forest surface in Catalonia. *B*. *sempervirens* is widely distributed in the study area, being essential for some of the ecosystem understory (because it is shade tolerant) but also frequent in open shrub areas. It is normally found in limestone of sub‐Mediterranean environments, although it can be also found in subalpine and Mediterranean forests covering a range of 100–1,900 m a.s.l. (Folch et al., 1984).

Catalonia has a high concentration of exotic species due to the high population density—and consequent trade movements and anthropization of the landscape—and the moderate climate conditions (Gassó et al., 2009). The box tree moth was first observed in Catalonia in 2014 (Bassols Isamat & Oliveras Giralt, 2014), but 2017 was the first year of high severity defoliation recorded specifically in northeastern Catalonia. Since then, the species has spread through the center of the territory.

### 2.3 General overview of modeling

**FIGURE 2:**
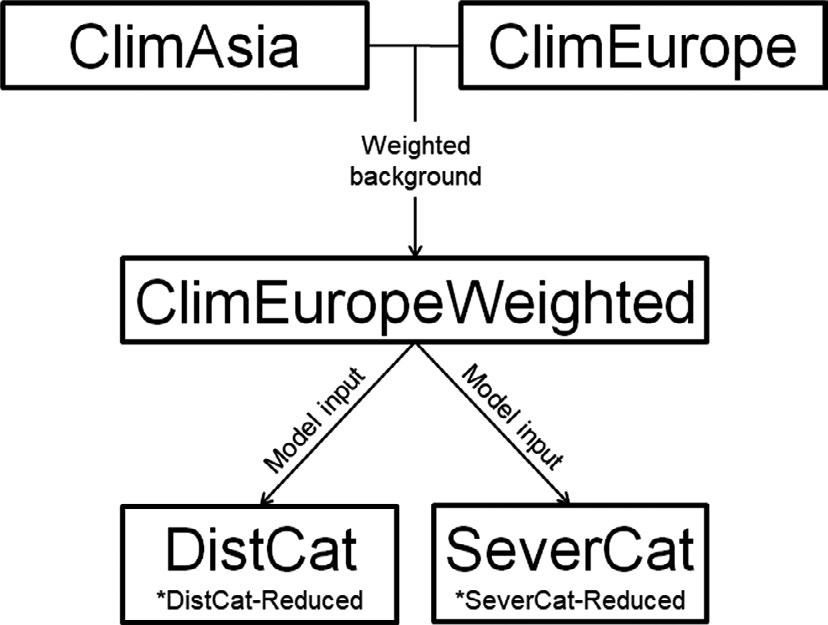
Conceptual diagram of the different models included in the study and the established relationship between them

The research presented in this paper comprises different interconnected models (Figure 2):

1. Climate suitability model for the distribution of *C*. *perspectalis* calibrated in the native area, Asia (ClimAsia). This model is projected to Asia and Europe.
2. Climate suitability model for the distribution of *C*. *perspectalis* calibrated in the invaded area, Europe (ClimEurope). This model is projected to Europe and Asia.
3. Climate suitability model for the distribution of *C*. *perspectalis* calibrated in the invaded area, Europe, but background information weighted with the ClimAsia projection in Europe (ClimEuropeWeighted). This model is projected to Europe and Asia.
4. Distribution model in Catalonia (DistCat). This is an ecological niche model that uses previous ClimEuropeWeighted output and other information as input variables. A variation of this model using fewer input variables was built (DistCat‐Reduced).
5. Severe defoliation model in Catalonia (SeverCat). This is an ecological niche model that uses previous ClimEuropeWeighted output and other information as input variables. A variation of this model using fewer input variables was built (SeverCat‐Reduced).

The above‐mentioned models are based on the SDM methodology (Guisan et al., 2017).

### 2.4 Climate suitability model approach

Climate suitability models for *C*. *perspectalis* in Asia and Europe were built combining *C*. *perspectalis* occurrences and associated environmental data. Occurrence data of *C*. *perspectalis* at the global level were obtained from the Global Biodiversity Information Facility (GBIF Backbone Taxonomy, 2019) and complemented with literature citations (Kim & Park, 2013; Wan et al., 2014). Information was accurately selected by discarding observations with incomplete information, without associated quality coordinates, spatially clustered records, and duplicated locations. A final set of 83 and 3,074 occurrence data were obtained from Asia and Europe, respectively. To calibrate the climate suitability model, 10,000 randomly generated background points were created in each model extent (ClimAsia extent: longitude = 95 to 150, latitude 20 to 45; ClimEurope extent: longitude = −15 to 50, latitude = 35 to 75; Elith et al., 2011; Phillips et al., 2009).

We regressed the presence/absence of *C*. *perspectalis* models for Asia and Europe with eight explanatory climate variables obtained from CHELSA (Karger et al., 2017) at 30 arc seconds (ca. 1,000 m) spatial resolution and mean values for 1979–2013 (Table 1). These eight variables were the final selection from an initial set of 19 climate variables that were subjected to a multicollinearity test using Pearson's correlation coefficient and variance inflation factor (VIF) analysis both in Asian and European regions (Marquaridt, 1970). We finally selected only one variable from each set of highly correlated variables (|*r*| > 0.7; VIF > 10) depending on its relative contribution to the overall model and corroborated by the described biological relevance to the studied species in the literature (Artola, 2019; Nacambo et al., 2014).

**TABLE 1:** Summary of variables used in each model

Variable	Type	Model
Mean diurnal range (bio2) [°C]	Bioclimatic	ClimAsia, ClimEurope, and ClimEuropeWeighted
Max temperature of warmest month (bio5) [°C]		
Min temperature of coldest month (bio6) [ºC]		
Annual precipitation (bio12) [mm/year]		
Precipitation seasonality (bio15) [coefficient of variation]		
Precipitation of driest quarter (bio17) [mm/quarter]		
Precipitation of warmest quarter (bio18) [mm/quarter]		
Precipitation of coldest quarter (bio19) [mm/quarter]		
Climatic suitability [%]		DistCat and SeverCat
Elevation [m]	Topographic	
Aspect [N‐W‐S‐E categories]		
Forest cover 1km radius [%]	Habitat composition	
Mean basal area at 1km radius [m^2^*ha^−1^]		
Habitat fragmentation (no landscape patches / no habitat classes)		
Habitat heterogeneity (nº landscape patches * nº habitat classes)		
*Buxus* *sempervirens* cover 10 km radius [%]		
Min distance to *Cydalima* *perspectalis* observations [m]	Dispersion	DistCat
Min distance to high severity *C*. *perspectalis* observations [m]		SeverCat

Climate suitability models for *C*. *perspectalis* in Asia and Europe were assembled by the species distribution modeling algorithm MaxEnt in R 3.6.1 (R Core Team, 2019), with the *dismo* (Hijmans et al., 2017) and the *maxnet* (Phillips, 2017) packages. MaxEnt is a machine learning algorithm used for describing probability distributions following the principle of maximum entropy, subject to restraints imposed by the presence of species and their surrounding environment (Phillips et al., 2009). Modifying MaxEnt algorithm parameters so that they are adequate for each study is highly recommended (Elith et al., 2011; Merow et al., 2013; Morales et al., 2017). Thus, according to the sample size, the broad extension, and the limited knowledge of the studied species, we parametrized the MaxEnt algorithm with β‐multiplier = 3, discarding the use of threshold prediction and featuring predictors with quadratic response curves. We realized a sample data‐splitting procedure for evaluating the model. First, models were calibrated with 70% of the initial data. Second, models were run 10 times taking the average as the final model. Finally, models were evaluated on the remaining 30% of initial data with two model evaluations: the area under the receiver operating characteristic curve (AUC; Hanley & McNeil, 1982) and the continuous Boyce index (CBI; Boyce et al., 2002). AUC values are between 0 and 1, where values close to 0.5 are equivalent to a random prediction; values greater than 0.8 are considered a reliable prediction, and values up to 1 represent perfect agreement of the model with the observed data (Fielding & Bell, 1997). CBI values are continuous between −1 and +1, where positive values indicate a model that presents predictions consistent with the distribution of occurrences in the evaluation; values close to zero indicate a model equivalent to a random model and negative values indicate counter predictions (e.g., predicting no occurrence in areas where actual presence is recorded; Hirzel et al., 2006; Manzoor et al., 2018).

Because invasive species are not in equilibrium with the invaded area, there are climatically suitable locations in which the species may not have arrived yet due to dispersal constraints and the invasion story. Hence, background sample considered in the model may include both “true” and “false” absences (Gallien et al., 2012). To analyze the adequacy of the models, we checked the ClimEurope model adequacy when projected in Asia and ClimAsia when projected in Europe (Srivastava et al., 2020). Finally, another SDM model called ClimEuropeWeighted was calibrated with occurrence data from Europe (the same that were used in ClimEurope) but background data were weighted with the ClimAsia projection in Europe. Weights for each background point (x) were obtained from the formula presented by Gallien et al. (2012):

$$\text{Weight}\left(x\right)=\frac{1}{1+{\left(\frac{\text{ClimAsia}\;\text{projection}\;\text{in}\;\text{Europe}(x)}{\text{ClimAsia}\;\text{projection}\;\text{in}\;\text{Europe}(x)‐1}\right)}^{2}}$$

The highest weight values (i.e., a low habitat suitability) were attributed to a background sample that showed a high level of agreement with the ClimAsia model projection (i.e., it probably represents a “true” absence). Comparison of these three approaches through respective output value correlations, AUC and CBI evaluations and climate variable response curves allowed us to analyze the data to define the distribution of the species in the native and invaded area.

### 2.5 Ecological niche model for distribution and severe defoliation

We built two models of potential distribution and severe defoliation (as an indicator of ecosystem impact) in Catalonia, where controlled quality data and distribution dynamics were more manageable than on a global scale. Occurrence data used in Catalonia were obtained from the community science project Alerta Forestal (AF), a collaborative tool where users upload field observation of forest disturbances (fire, drought, or some forest pests) providing a picture, its coordinates, and the level of disturbance severity (low, moderate, high, and very high according to the percentage of defoliation). We combined AF data with observations obtained from forest rangers (Agents Rurals de la Generalitat de Catalunya) in 2018 and 2019 that sampled boxwood locations all along the study area (avoiding sampling bias) and also reported the level of disturbance severity (four levels according to the percentage of defoliation, equivalent to AF data). Data quality was checked by discarding observations with no associated quality coordinates, wrong locations, or species identification and spatially clustered records. Disturbance severity levels were subsequently evaluated by experts reviewing the percentage of defoliation using the picture facilitated. Two final sets of 566 and 1,022 occurrence data points were collected in Catalonia during 2018 and 2019, respectively. We generated a random located set of 10,000 background points in Catalonia to calibrate the models.

Potential distribution and severity were defined with the use of biotic and abiotic variables (Table 1), which were selected according to ecological descriptions of the species (Artola, 2019; Nacambo et al., 2014; Roura‐Pascual et al., 2012). Abiotic variables were defined by elevation and aspect variables obtained from the Digital Elevation Model of the Spanish National Geographic Institute (Ministerio de Fomento, 2011) as a topographic approximation and the ClimEuropeWeighted model projection as a summary of relevant climate information.

Biotic determinants were defined by the amount of suitable habitat composition for the species, as they are demonstrated to improve species distribution modeling (Meier et al., 2010; Stanton et al., 2012). First, we calculated the percentage of forest and the mean basal area at a 1 km radius around every observation plot, obtained from *Variables* *Biofísiques* *de* *l’Arbrat* *de* *Catalunya* (Bozal & Orriols, 2016). Second, we calculated the habitat fragmentation and habitat heterogeneity from the number of patches and habitat classes per square kilometer considered in the Land Cover Map of Catalonia (Ibàñez et al., 2002). We finally included the percentage of the host species *B*. *sempervirens* at a 10 km radius around each plot (i.e., the reported maximum flight distance per year for *C*. *perspectalis* adults; Bras et al., 2019; Straten & Muus, 2010). Boxwood presence was defined by the combination of occurrences reported in the Fourth Spanish National Forest Inventory (IV IFN, Ministerio de Agricultura Pesca Alimentació y Medio Ambiente 2008–2017) and the Map of Habitats of Catalonia (Carreras, 2004; Carreras et al., 2014).

To define the dispersal capability of the species, we calculated the distance between each 2019 species observation and the closest 2018 observation. For defoliation severity dynamics of *C*. *perspectalis* (SeverCat model), we repeated the same methodology but selected only high severity observations of 2018 and 2019 and calculated the minimum distance to such points. All variable information for SeverCat and DistCat models was prepared at a 1 km pixel resolution.

The potential distribution model was calibrated with the occurrence data from 2019 and climatic, topographic, habitat composition, and map of distances for 2018 as explanatory variables, using the MaxEnt algorithm in R 3.6.1 (R Core Team, 2019), with the *dismo* (Hijmans et al., 2017) and the *maxnet* (Phillips, 2017) packages. The severity defoliation model was also calibrated with occurrence data from 2019 and the same explanatory variables but using map of distances to high severity points for 2018. Once again, models were calibrated with 70% of initial data and evaluated with the remaining 30% and AUC and CBI validation.

The distribution of *C*. *perspectalis* may be limited to the distribution of its host plant and dispersal story (i.e., distance between observations). To analyze the relevance of the other variables, we generated the models DistCat‐Reduced and SeverCat‐Reduced that were repetitions of previous models but using a new background set randomly distributed in a 10 km buffer around each presence (thus eliminating the distance factor; Gallien et al., 2012; Phillips et al., 2009) and masking only the locations with the presence of boxwood (thus eliminating this factor).

## 3 RESULTS

### 3.1 Dealing with nonequilibrium in the invaded area

**FIGURE 3:**
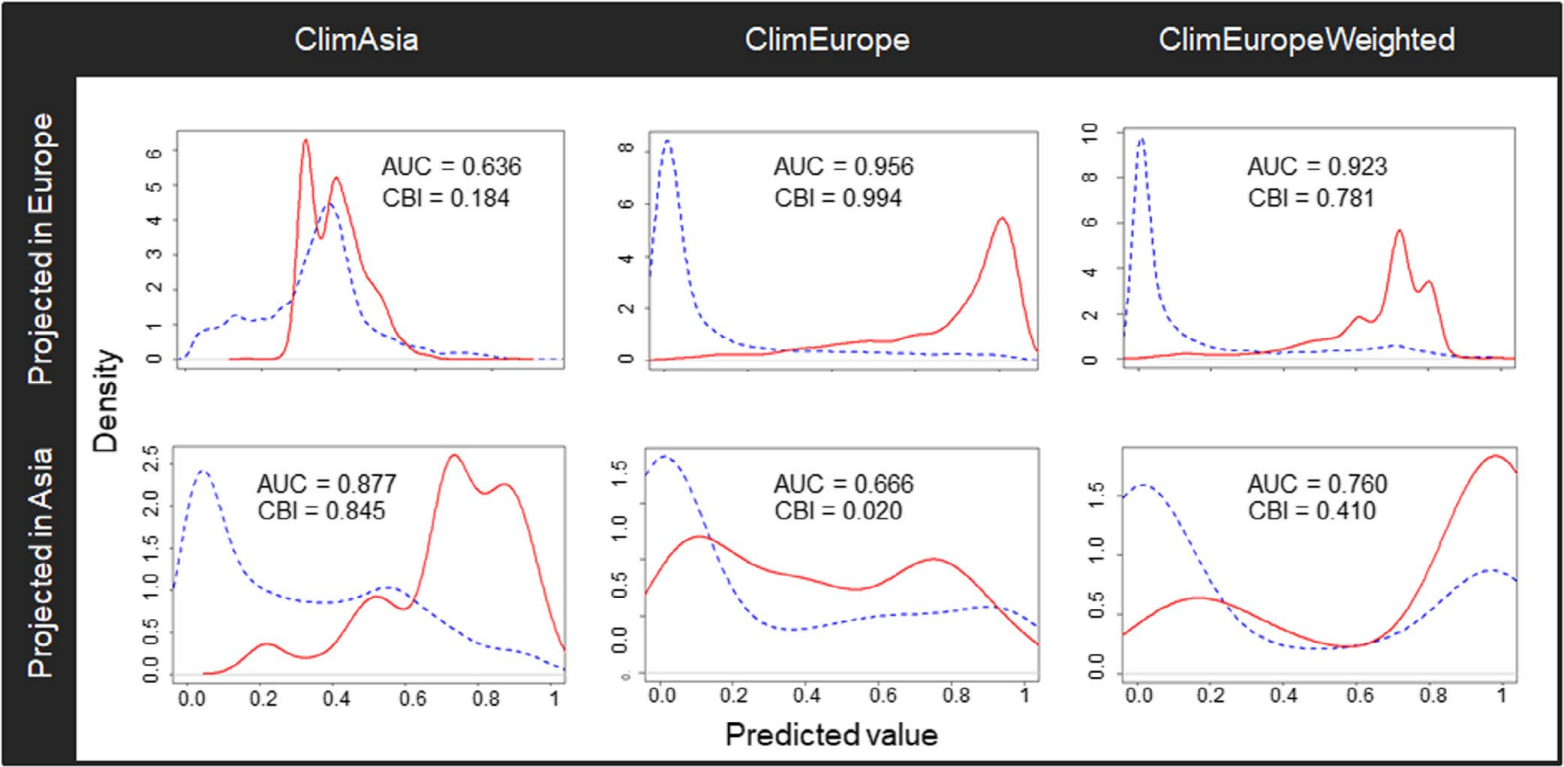
The density of predicted values for background sample (blue slash) and occurrences (red line) according to each climate model projected in Asia and Europe. The area under the receiver operating characteristic curve (AUC) and continuous Boyce index (CBI) scores are added to each model and projection

Three approaches were used to evaluate the adequacy of the data defining *C*. *perspectalis* distribution in the invaded area. The ClimAsia model had an AUC of 0.88 and a CBI of 0.85 in the native area, but 0.64 and 0.18, respectively, when projected to Europe (Figure 3). The ClimEurope model achieved good performance when projected in Europe (AUC = 0.96 and CBI = 0.99), but its evaluation fell to 0.67 and 0.02, respectively, when applied to Asia. Finally, the ClimEuropeWeighted model scored an AUC of 0.92 and a CBI of 0.78 in Europe and 0.76 and 0.41, respectively, when projected to Asi

a.

**FIGURE 4:**
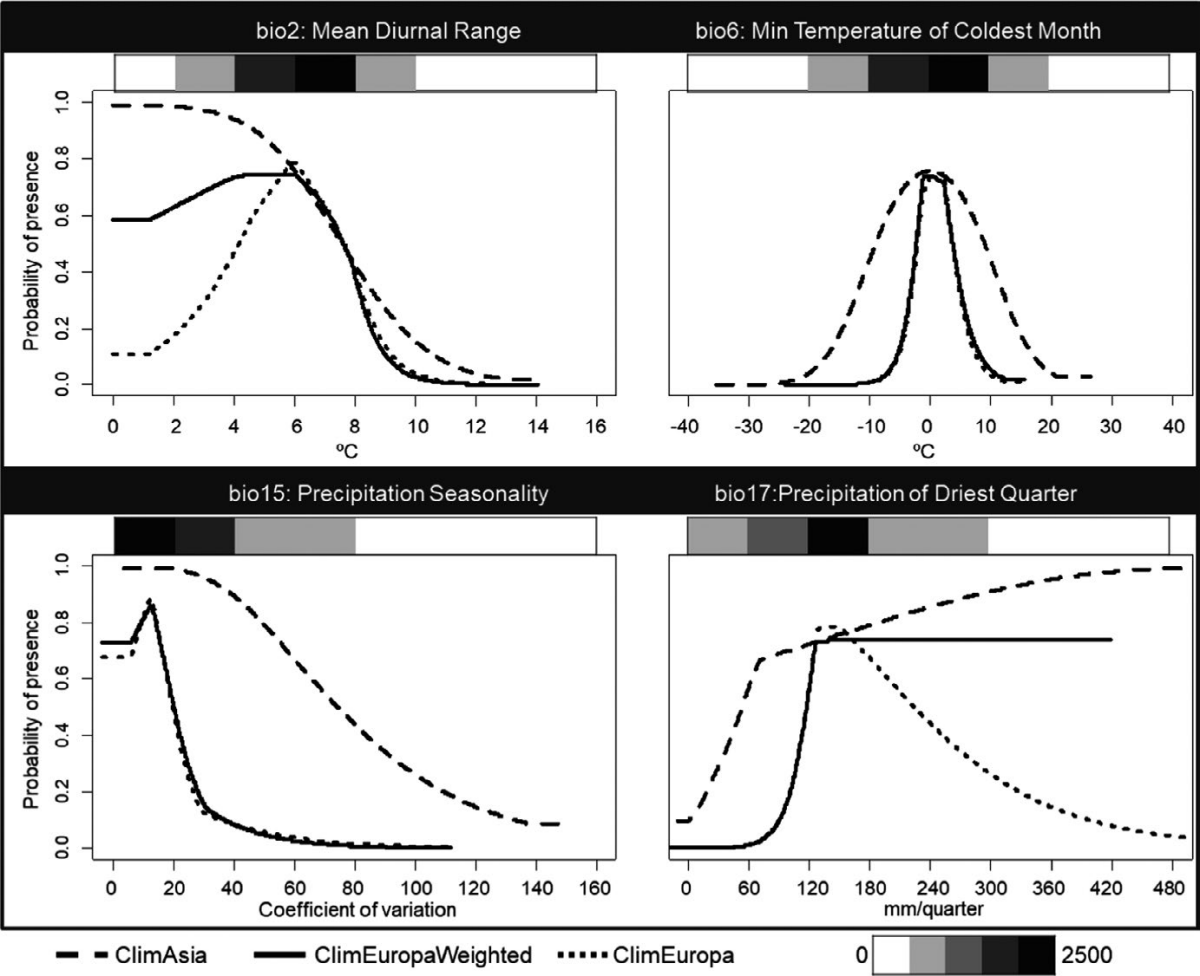
Response curves showing how the four main environmental variables affects each climate model prediction for *Cydalima* *perspectalis* W. These response curves are the mean of 10 runs performed for each model. Gray heatmaps indicate the number of real observations found in every value range

When analyzing the response curves for the main variables, similar variable contributions were found in the different models. However, in general, ClimAsia accounted for a wider range of values for each variable than ClimEurope, while ClimEuropeWeighted was in between the other two (Figure 4).

### 3.2 Climate suitability in the native and invaded areas

Heuristic estimation of the relative contribution of each climate variable defined Precipitation of driest quarter (bio17), Min temperature of coldest month (bio6), Precipitation seasonality (bio15; i.e., the temporal distribution of precipitation on a monthly basis), and Mean diurnal range (bio2; i.e., the annual mean of the difference between the maximum and minimum daily temperature) as the main explanatory variables both in ClimAsia and ClimEurope (Figure 4). These four variables combined accounted for 87.6% and 94.3%, respectively, of the variability in the climatic models. According to the model, the most suitable areas for *C*. *perspectalis* are those with precipitations over 90 mm in the driest quarter and low coefficient of variation of precipitation during the year. Mild temperatures (annual minimal between −2 and 2ºC) and low diurnal variation (lower than 12ºC) may favor the presence of *C*. *perspectalis*.

**FIGURE 5:**
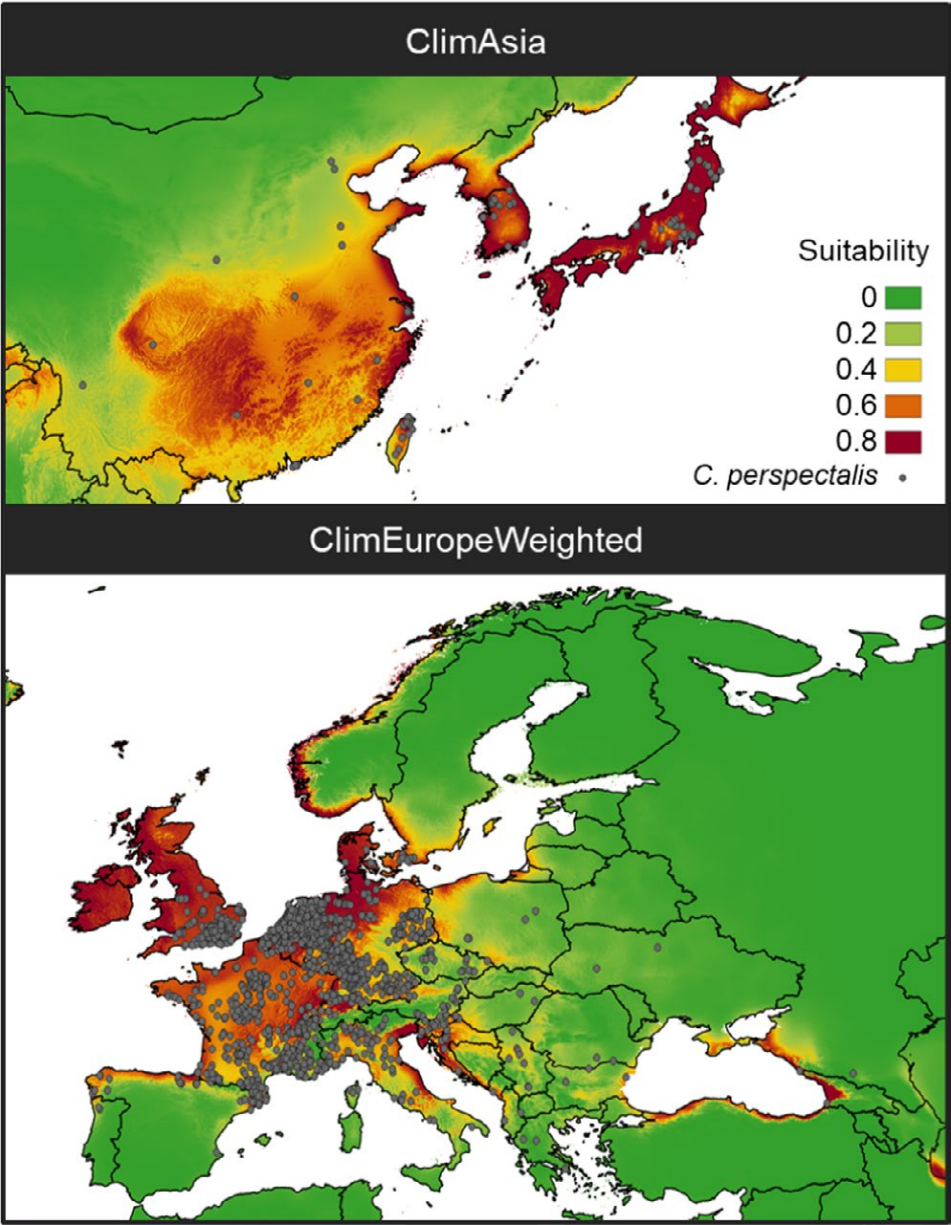
Maps of climate suitability for *Cydalima* *perspectalis* W. in the native area (according to the ClimAsia model) and the invaded area (according to the ClimEuropeWeighted). Gray points indicate known species occurrences while color legend indicates the suitability index

These variables defined the area of high suitability for *C*. *perspectalis*, including southeastern China, Taiwan, Japan, and North and South Korea in the native area (Figure 5). There is an apparent trend toward continentality for both the Asian and European species distributions. In the invaded area, central European countries including the United Kingdom were the main suitable areas for the species (Figure 5). Here again, in the Mediterranean Sea, the Cantabrian Sea, the Black Sea, the Baltic Sea, and the North Sea continentality seemed to be relevant. The main mountain ranges such the Alps, the Pyrenees, the Balkans, and the Carpathian were excluded as suitable areas for the species.

### 3.3 Ecological niche

**FIGURE 6:**
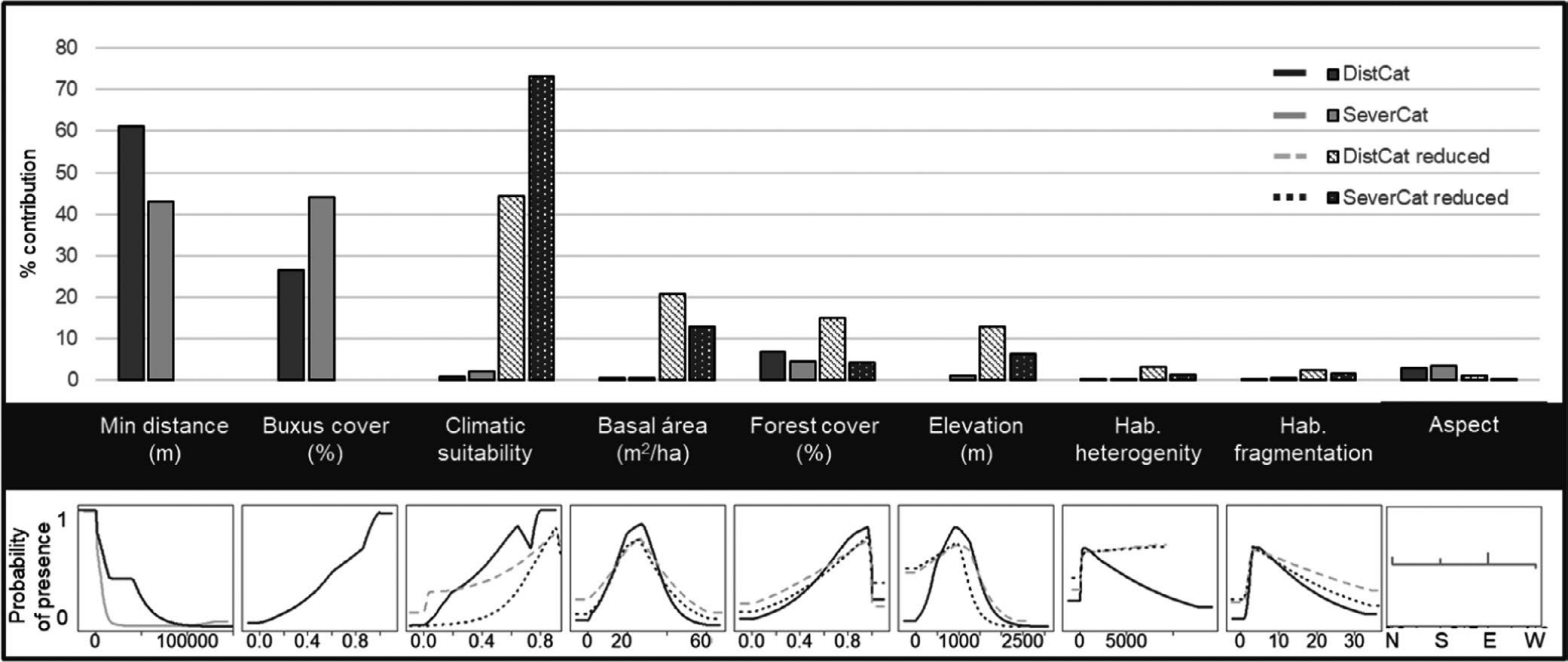
Estimates of the relative contribution and response curve of each ecological variable to each ecological niche model for *Cydalima* *perspectalis* W. in Catalonia

**FIGURE 7:**
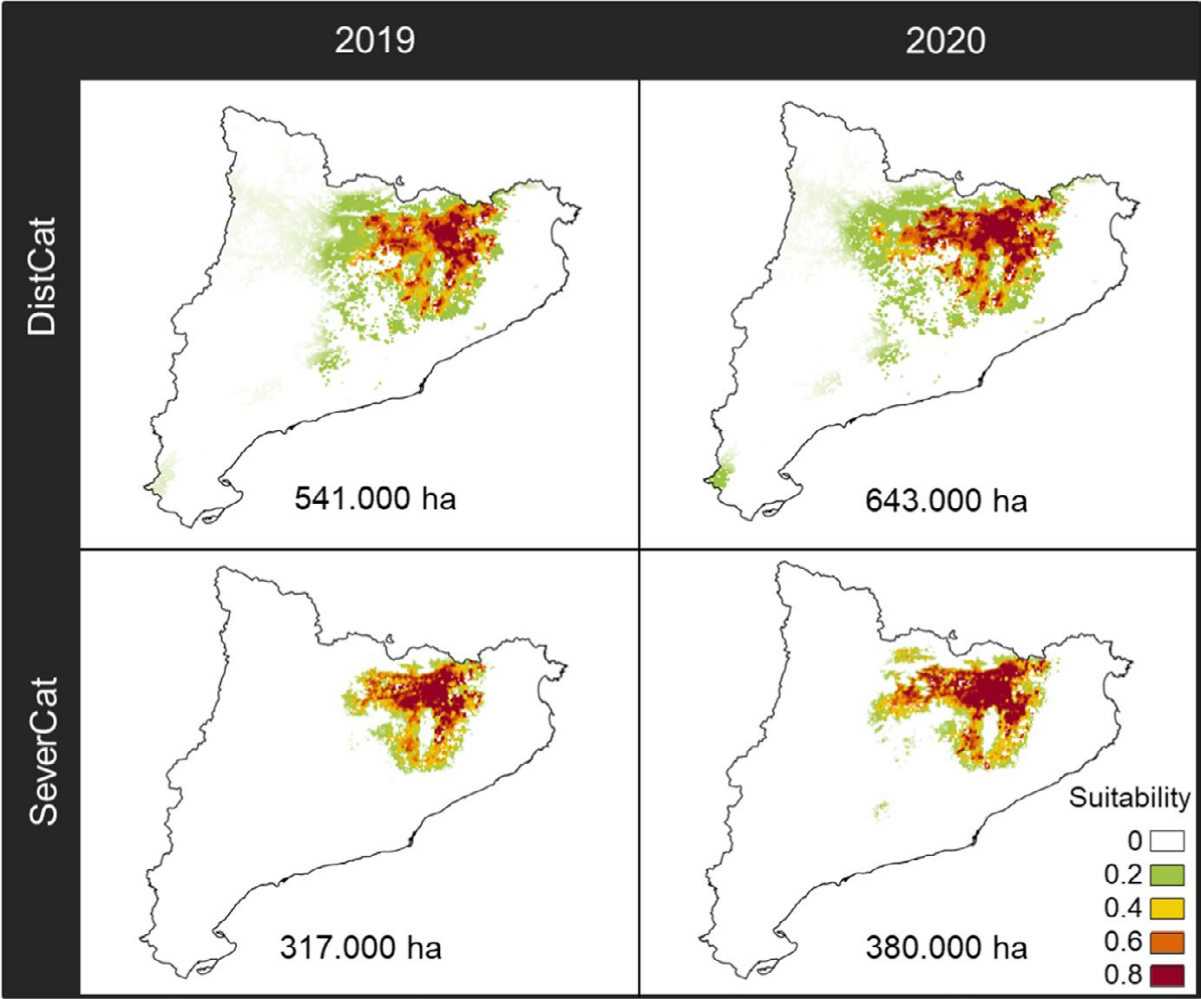
The distribution model (DistCat) and the severe defoliation model (SeverCat) including areas affected for *Cydalima* *perspectalis* W. in Catalonia for 2019 and projected to 2020. Maps are masked with the *Buxus* *sempervirens* L. distribution

Ecological niche models for *C*. *perspectalis* in Catalonia showed good adjustment with the observed data (DistCat, AUC = 0.90 and CBI = 0.92; SeverCat, AUC = 0.89 and CBI = 0.90). Boxwood cover and Min distance to *C*. *perspectalis* observations in the previous year were the two main variables that explained the distribution of the species and severe defoliation (Figures 6 and 7). These two variables combined accounted for 86% and 87.2% of the variability of the DistCat and SeverCat models, respectively, diminishing the effect of other variables. Response curves indicated that the probability of presence of the species and the severity of defoliation increased almost linearly with the increase in the boxwood cover, while this probability decreases as Min distance to *C*. *perspectalis* observations were longer than 15 km for DistCat and 40 km for SeverCat.

The execution of the DistCat‐Reduced and SeverCat‐Reduced models revealed the role of other variables beyond the presence of boxwoods and the distance between observations of *C*. *perspectalis*. Both models showed good adjustment with the observed data (DistCat‐Reduced, AUC = 0.75 and CBI = 0.68; SeverCat‐Reduced, AUC = 0.80 and CBI = 0.74). Climate suitability was the main variable in both models, followed by mean basal area and the forest cover. Elevation was marginally relevant while aspect, habitat heterogeneity, and habitat fragmentation variables did not provide additional information to the model (Figure 6).

### 3.4 Regional distribution and severe defoliation

Although comparison of models for the distribution of *C*. *perspectalis* (DistCat) and the severity of defoliation (SeverCat) had similar patterns both in the contribution of the explanatory variables and in the resulting maps, some differences must be noted (Figure 6). Boxwood cover relevance in the DistCat model was half that of the contribution of distance between observations (27% and 59%, respectively). Both variables were similarly relevant in the SeverCat model (44% and 43%, respectively). The additional contribution of other variables was minimal in both models. Ignoring boxwood cover and distance between observations, the SeverCat‐Reduced model was more closely related to the climate suitability variable (73%) than any habitat composition factors (none higher than 13%). In addition, the SeverCat‐Reduced model revealed an exponential relationship with climate suitability variable, increasing the severe defoliation probability when the climate suitability value was higher than 0.5 (Figure 6). By contrast, variable contributions in the DistCat‐Reduced model were more balanced (44% for the climate suitability variable, 21% for basal area, and 15% for forest cover), and the relationship between model performance and the climate suitability variable was almost linear from 0 to 1. Additional contribution of aspect and habitat fragmentation and heterogeneity variables were poor in all four models.

**FIGURE 8:**
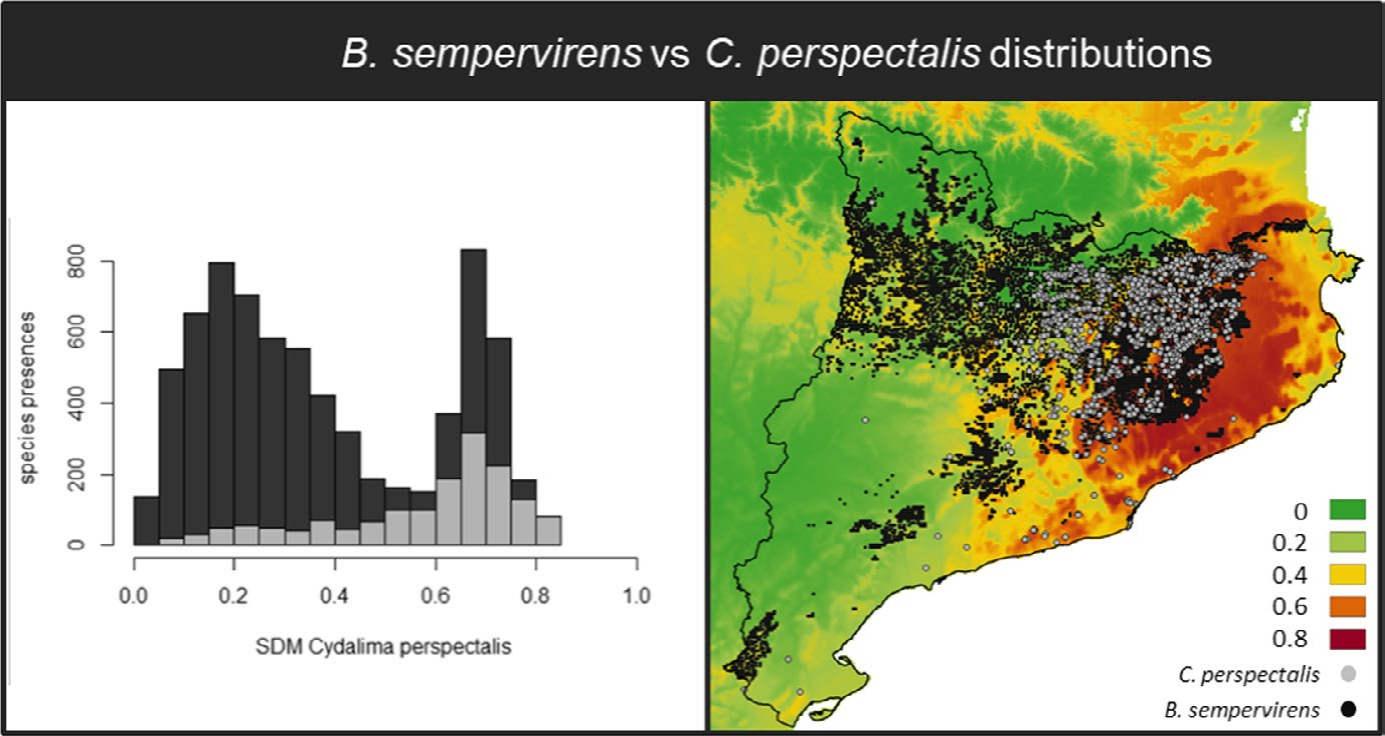
Spatial synchrony between *Buxus* *sempervirens* L. distribution (dark gray) and *Cydalima* *perspectalis* W. distribution (light gray) according to climate suitability (color index on the right panel)

Observations of *C*. *perspectalis* in 2018 in Catalonia occurred in the eastern interior of the region, while observations in 2019 expanded further west and south (Figure 8, right panel). Thus, the DistCat and SeverCat models for 2019 and their projections for 2020 produced maps with this same trend toward the western portion of the territory. Our models defined 541,000 ha of the distribution area and 317,000 ha of severe defoliation for *C*. *perspectalis* in 2019, while projections to 2020 predicted 643,000 ha and 380,00 ha, respectively (Figure 7). These areas remain strongly related to the spatial coincidence of the boxwood distribution area with locations of high climate suitability for *C*. *perspectalis* (Figure 8).

## 4 DISCUSSION

### 4.1 Realized regional niche versus global niche

The application of SDMs to invasive species is controversial because invasive species are still expanding and not in equilibrium with the novel environment (Gallien et al., 2012). To address this issue, we compared three climate models considering realized regional niches (range of conditions actually occupied by a given species) in the native area (ClimAsia) and the invaded area (ClimEurope) and the combination of both, estimating the global niche (ClimEuropeWeighted; Gallien et al., 2012; Roura‐Pascual et al., 2009; Uden et al., 2015).

Climate models developed from occurrence data in Asia (ClimAsia) and Europe (ClimEurope) resulted in good quality adjustment in respective calibration areas but were not reliable on a worldwide scale. Differences between these two models were explained because the presence in Asia covered a wider range of values in the considered climate variables and, therefore, presented a wider climate niche than ClimEurope. This is a case of niche shift in biological invasion, and it is widely discussed in distribution and invasion studies (Zhang et al., 2020). Niche variation may correspond to the fact that the species in its native area had more time to expand and to be in pseudo‐equilibrium with the environment (Gallien et al., 2012), although the small number of occurrences collected in Asia for this species may increase such an effect (Hernandez et al., 2006; Wisz et al., 2008). Consequently, the ClimAsia model applied to Europe did not provide a good match because it considered many false positives (causing specificity problems). Moreover, although the ClimEurope model provided good adjustment in Europe, it overestimated false negatives when analyzed globally (causing sensitivity problems). Thus, using only one estimate of realized regional niches (the native or the invaded area) may misrepresent the environmental preferences of the species and may lead to incomplete predictions (Broennimann et al., 2012; Fitzpatrick et al., 2006).

The combination of information through the realized regional niche background sample weighted with the global niche information increased prediction accuracy and thus limited the influence of false negatives, maintaining the true positives of the regional model and letting climate variables at the regional level refine the model. Thus, the ClimEuropeWeighted model perfectly matched occurrences in Europe and estimated that the niche of *C*. *perspectalis* may be slightly wider than the realized niche. Hence, the species still has space for expansion.

### 4.2 Ecological niche

In this study, we have presented a modeling design that considered the dispersal limitations of the species, the biotic environment (including community constrains), and the abiotic environmental conditions (including climate suitability) because the combination of these influences determines species occurrence in a given space area (Lortie et al., 2004; Sóberon, 2007). Dispersal processes were approximated with distance between observations; this variable accounted for the species distribution and impact as identified by the DistCat and SeverCat models. *C*. *perspectalis* observations were usually grouped, normally not exceeding a 15 km distance. This aggregation pattern is common in invasive species, which increase their radius of dispersal more rapidly in the early years of invasion (Roques et al., 2016). However, human‐induced dispersion is a critical factor in the distribution of *C*. *perspectalis* and may facilitate a faster dispersal rate than estimated by the models.

As expected, boxwood cover was also a key factor for understanding the species distribution and severe defoliation. *C*. *perspectalis* is totally dependent on boxwood as a host species and does not develop on other host plants (Straten & Muus, 2010). Thus, this biotic factor may limit the distribution of *C*. *perspectalis* but at the same time accentuate the risk for the boxwood in the territory. In addition, boxwoods are usually used in gardening (parks, cemeteries, private gardens, etc.) and may play a key role of connectors between boxwood forests. Other biotic factors (habitat descriptors in terms of fragmentation and heterogeneity) were considered but were not relevant for the distribution or severity of *C*. *perspectalis*. These findings indicate that this species is a nonhabitat specialist, similar to other species (Litvinchuk et al., 2014; Sardà‐Palomera & Vieites, 2011; Vallecillo et al., 2009). Forest cover and mean basal area were also considered as habitat descriptors as well as for their influence on microclimatic conditions (direct radiation, forest temperature, etc.) that may affect insect development (Notter‐Hausmann & Dorn, 2010). Species distribution was sensitive to these factors, performing a better response in areas of high forest cover and basal area between 20 and 30 m^2^/ha, and this may suggest that *C*. *perspectalis* performs better on understory boxwood than on open shrub areas. However, such a relation should be specifically questioned because observations and severe defoliation by the species were observed in both habitat types as confirmed by Danés et al. (2020) and Artola (2019).

Abiotic environmental conditions were considered through climate suitability and the two topographic variables. The use of climate suitability as a model input summarizing all climate information is increasingly relevant in ecological research (Jaime et al., 2019). Although climate suitability was not related to the species distribution and did not impact the DistCat and SeverCat models, it was the main factor when distance between observations and boxwood cover was excluded (i.e., climate is critical once the species arrives in a boxwood area). Climate models described *C*. *perspectalis* suitability through mild climate conditions, with low precipitation seasonality combined with a small temperature diurnal range. The relevance of those climatic variables has also been emphasized by other studies that identified climate conditions during the larval development as the key for the species success (Nacambo et al., 2014; Suppo et al., 2020). For example, the mean diurnal range may lead to a high degree day value (warmth above a development threshold temperature); this factor is highly related to insect development, as observed by Herms (2004) and Nacambo et al. (2014). Mild temperatures (minimum around 0) and low precipitation seasonality directly influence insect activity, growth, and phenology (Jaworski & Hilszczański, 2014). In addition, low precipitation may indirectly affect the insect development by damaging the host plant and, thus, resource availability (Jaworski & Hilszczański, 2014; Zhu et al., 2014), but such interaction should be specifically studied for these species.

Exposed climate conditions defined the climate of Western Europe and the British Isles (where precipitation during the dry period is over 200 mm/quarter) as well as the Atlantic Ocean, the Black Sea, the North Sea, and the Mediterranean coasts (where there is little precipitation and temperature seasonality; Karger et al., 2017). Thus, climate suitability for *C*. *perspectalis* was characterized by a certain continentality effect, being more suitable in the coast than inland, both in its native and invasive area (Figure 5); these findings had been identified by Nacambo et al. (2014). Although the elevation variable was not significant in our model, climate conditions defined lower climate suitability at higher elevations. The model indicates climatically suitable areas where the species is already found and areas where it has not been established (Ireland, Scotland, the coast of Norway, and Sweden, etc.) but potentially could subject to the presence of boxwoods.

Finally, because precipitation seasonality and temperature are estimated to increase with climate change in the areas where the species is currently found (Karger et al., 2017), our results suggest that future conditions may affect the success of *C*. *perspectalis* (proposed in terms of phenology; Suppo et al., 2020) or even compromise its expansion and severity. Thus, the positive contribution of climate change on alien species performance is unclear (Bebber et al., 2013; Peterson et al., 2008).

### 4.3 Invasion stages: dispersion and impact

A description of the process by which *C*. *perspectalis* has invaded Catalonia is crucial to understand the invasion stage of the species (following the progress of arrival, establishment, dispersion, and impact). *C*. *perspectalis* has been continuously present in Catalonia since 2014, indicating that the arrival and establishment phases have been reached. Modeling distribution dynamics (as a proxy of dispersion) and defoliation severity (as a proxy of impact) separately allowed us to differentiate the mechanisms behind the two processes. Although both models showed high sensitivity to the same variables, climate suitability contributed more to the impact model (SeverCat), so this may be a mitigating factor for severe defoliation impact (i.e., the species could spread to different localities, but damage to the boxwoods would be severe only where there is higher climate suitability).

The output model maps indicated that areas of distribution and severe defoliation were linked: the higher the probability of occurrence of the species, the higher the defoliation impact (Figure 7). Moreover, projections for 2020 indicated that potential areas to invasion and severe defoliation may increase at a similar rate. However, climate constraints to severe defoliation combined with the continentality pattern of climate suitability may reduce the inland advance of severe defoliation impact. This may cause slight asynchrony between the distribution and severe defoliation areas, as some occurrences were found far from the area of severe impact (Figure 7), but pooled data from 2 years are not enough to check such patterns.

Natural enemies such as birds, bats, other insectivores, or parasitoids may play an important role controlling the distribution of *C*. *perspectalis*, as happens with other invasive species (Snyder & Evans, 2006; Wanger et al., 2011). Wan et al. (2014) described the natural enemies of *C*. *perspectalis* in Asia, but in Europe only a few studies have covered this topic. Authors have described predation of *C*. *perspectalis* by *Passer* *domesticus* L., *Parus* *major* L., *Turdus* *merula* L., and *Ficedula* *albicollis* Tem (Bakay & Kollár, 2018). Moreover, *C*. *perspectalis* may interact with biotic diseases like fungi *Cylindrocladium* sp. (Henricot et al., 2000) altering both impacts on *B*. *sempervirens*, although this topic has not been studied to the date.

### 4.4 Impact and management

Consecutive defoliation periods of boxwoods by *C*. *perspectalis* may lead to host plant decay or death. Although boxwood can resprout after defoliation attacks, the synchrony between the generation of new leaves and the emergence of new insect generations (3–5 generations per year as mentioned above) compromises the viability of boxwoods (Artola, 2019; Straten & Muus, 2010). Boxwood loss has cultural, economic, and ecological consequences (Mitchell et al., 2018). Ecologically, boxwoods host hundreds of species; indeed, 63 of them are found only in this host plant (Mitchell et al., 2018). At the landscape scale, boxwoods represent a key element of different Mediterranean habitats, both as an understory species and as dominant species in scrublands (Di Domenico et al., 2012). In Catalonia, boxwoods are abundant in 30% of the forest area (Carreras, 2004; Danés et al., 2020) and are present in some singular forests (Comas et al., 2013). The loss of understory boxwoods may alter community dynamics by reducing inter‐specific competition for soil water consumption or via fuel changes (in terms of both quantity and continuity) that could modify the fire regime in particular areas.

In the present study, climate suitability was described as an important variable in *C*. *perspectalis* distribution, but especially when defining high defoliation area, as mentioned in the previous section. Our results suggest that climate suitability is subject to a continentality effect (less suitability inland) and does not co‐occur with boxwood distribution (Figure 8). Thus, those boxwoods placed in areas with less favorable climatic conditions for the insect (i.e., inland and in high elevation) would be safe from high defoliation.

The impact of *C*. *perspectalis* on boxwoods concerns land managers. This study emphasizes that two key factors in the species distribution and impact are insect dispersal capability and climate suitability (considering boxwood presence). Because *C*. *perspectalis* cannot travel long distances by itself, the control of human activities (via commercial trades or via accidental anthropochorous transfer between forest areas) is crucial to limit the rapid expansion of the species. On the other hand, climate limitations have defined those areas most vulnerable to severe defoliation and, therefore, where protection efforts may be targeted. At the same time, the cultural value of particular forests should be valued to prioritize those singular areas of the territory.

Finally, it is necessary to question what the future of *C*. *perspectalis* in the invaded areas will be. When *C*. *perspectalis* impacts boxwoods in climatically suitable areas, its viability could be compromised due to food shortages. This could lead to a fatal decline in the population of the insect or to a cyclical relationship between boxwood defoliation–resprout and moth population dynamics. Hence, we emphasize the need for specific research that studies the viability of *C*. *perspectalis* and its host plant in the future.

## 5 CONCLUSIONS

In this study, we found that the distribution of *C*. *perspectalis* mainly depends on the presence of its host plant and its ability to spread, so in the near future (with enough time for *C*. *perspectalis* to fully spread), all boxwoods might be affected by the insect. However, climate suitability may be a handicap for *C*. *perspectalis* development in some areas. The climatic niche is characterized by limited seasonality of precipitation and temperatures and low diurnal ranges, defining higher suitability in Western Europe and in coastal areas. The case of *C*. *perspectalis* may not be favored by variations due to climate change, but climate niche projections would be necessary. Habitat descriptors and topographic variables were not relevant in our models; hence, this species is not habitat selective. However, the species is not in pseudo‐equilibrium with the invaded environment, so the processes of interaction with new competitors and predator species are still unknown.

Understanding the invasion phases in the area of invasion is essential to differentiate the processes of dispersion and impact. We found that a high climate effect markedly impacts the process, indicating that in some areas where the species could spread, its impact may be mitigated due to unsuitable climatic conditions. This information is valuable when designing management plans, but questions remain regarding the boxwood resprout capability after defoliation or how *C*. *perspectalis* may interact with other disturbances.

## CONFLICT OF INTEREST

The authors of this study declare no conflicts of interest.

## AUTHOR CONTRIBUTIONS

**Quim Canelles:** Conceptualization (lead); Data curation (lead); Formal analysis (lead); Investigation (lead); Methodology (lead); Software (lead); Validation (lead); Writing‐original draft (lead); Writing‐review & editing (equal). **EMILI Bassols:** Conceptualization (equal); Data curation (equal); Investigation (equal); Writing‐review & editing (equal). **Jordi Vayreda:** Conceptualization (equal); Investigation (equal); Writing‐review & editing (equal). **Lluis Brotons:** Conceptualization (lead); Funding acquisition (lead); Investigation (equal); Supervision (lead); Writing‐review & editing (equal).

### OPEN RESEARCH BADGES

This article has earned an Open Data Badge for making publicly available the digitally‐shareable data necessary to reproduce the reported results. The data is available at (https://www.gbif.org/; https://chelsa‐climate.org/) or via request (http://www.alertaforestal.com/; http://agricultura.gencat.cat/ca/inici).

## Data Availability

Data are available in open repositories (https://www.gbif.org/; https://chelsa‐climate.org/) or via request (http://www.alertaforestal.com/; http://agricultura.gencat.cat/ca/inici). R Code is available at https://github.com/quimcanellestrabal/Cydalima_QuimCanelles.
